# Long noncoding RNA FAM157C contributes to clonal proliferation in paroxysmal nocturnal hemoglobinuria

**DOI:** 10.1007/s00277-022-05055-8

**Published:** 2023-01-06

**Authors:** Honglei Wang, Hui Liu, Liyan Li, Yingying Chen, Zhaoyun Liu, Lijuan Li, Shaoxue Ding, Kai Ding, Rong Fu

**Affiliations:** Department of Hematology, Tianjin Medical University General Hospital, 154 Anshan Street, Heping District, Tianjin, 300052 China

**Keywords:** Paroxysmal nocturnal hemoglobinuria, PNH clone, LncRNAs, Proliferation, FAM157C

## Abstract

Paroxysmal nocturnal hemoglobinuria (PNH) is a rare clonal disease of hematopoietic stem cells (HSCs). Long noncoding RNAs (lncRNAs) perform a wide range of biological functions, including the regulation of gene expression, cell differentiation, and proliferation, but their role in PNH remains unclear.

CD59^−^ and CD59^+^ granulocytes and monocytes from 35 PNH patients were sorted. High-throughput sequencing was analyzed in 5 PNH patients, and differentially expressed lncRNAs and mRNAs were identified. The mRNAs with fragments per kilobase of exon model per million mapped fragments (FPKM) > 10 in at least 3 patients were selected, and experiments were performed to identify their upstream regulatory lncRNAs. The expression of selected mRNAs and lncRNAs was verified by qRT‒PCR, and the correlation of these expression patterns with clinical data from other 30 PNH patients was analyzed. Then, the functions of the lncRNAs were studied in the PIGA-KO-THP-1 cell line.

Transcription analysis revealed 742 upregulated and 1376 downregulated lncRNAs and 3276 upregulated and 213 downregulated mRNAs. After deep screening, 8 highly expressed mRNAs that were related to the NF-κB pathway were analyzed to determine coexpression patterns. LINC01002, FAM157C, CTD-2530H12.2, XLOC-064331 and XLOC-106677 were correlated with the 8 mRNAs. After measuring the expression of these molecules in 30 PNH patients by qRT‒PCR, lncRNA FAM157C was verified to be upregulated in the PNH clone, and its expression levels were positively correlated with the LDH levels and CD59^−^ granulated and monocyte cell ratios. After knockdown of the FAM157C gene in the PIGA-KO-THP-1 cell line, we found that the cells were arrested in the G0/G1 phase and S phase, the apoptosis rate increased, and the cell proliferation decreased.

LncRNA FAM157C was proven to promote PNH clone proliferation, and this is the first study to explore the role of lncRNAs in PNH.

**Supplementary Information:**

The online version contains supplementary material available at 10.1007/s00277-022-05055-8.

## Introduction

Paroxysmal nocturnal hemoglobinuria (PNH) is an acquired clonal disorder of hematopoietic stem cells (HSCs) that is caused by somatic mutation of the phosphatidylinositol glycan A (PIG-A) gene, which is located on chromosome Xp22.1. PIGA mutation leads to impaired glycosylphosphatidylinositol (GPI) synthesis, resulting in the loss of GPI-anchored protein (GPI-AP) on the cell surface (such as CD59 and CD55) and the disruption of the increased sensitivity of blood cells to complement activation; the main clinical manifestations of PNH are chronic intravascular hemolysis, bone marrow failure, and high risk of thrombosis [1–3]. The PIG-A mutation alone is necessary but insufficient to explain clonal expansion in PNH. PNH clones do not self-renew and survive for only 3–4 months [4]. To cause PNH, PIG-A mutation must result in proliferative advantages. However, the mechanism underlying the proliferative advantages of PNH clones has remained unclear to date.

Long noncoding RNAs (lncRNAs) range from 200 nt to ~ 100 kb in length; lncRNAs do not encode proteins and are located in the nucleus or cytoplasm [5, 6]. It is now well recognized that more than 75% of the human genome is functional and encodes large numbers of LncRNAs [7]. LncRNAs perform a wide range of biological functions, including the regulation of gene expression, such as chromosome dosage compensation, imprinting, epigenetic regulation, nuclear transport, transcription, mRNA splicing and translation, as well as cell differentiation, cell proliferation and substance metabolism [8, 9]. In the past decade, the abnormal expression of LncRNAs has been proven to be involved in the pathogenesis of many diseases, including tumors, metabolic diseases and cardiovascular diseases [10–12]. At present, studies on the mechanism underlying PNH clone proliferation have mainly focused on protein-coding genes, and the function and clinical significance of lncRNAs in PNH remain unknown. The purpose of this study was to investigate the role of lncRNAs in PNH clone proliferation.

## Methods

### Patients and clinical samples

A total of 35 PNH patients who were diagnosed in the Hematology Department of Tianjin Medical University General Hospital from November 2017 to August 2019 according to international PNH Study Group Criteria were enrolled in our study [13]. High-throughput sequencing was analyzed in 5 PNH patients. Then, selected lncRNA and mRNA expression levels were verified in other 30 PNH patients, and the correlations of these expression levels with clinical indexes, including hemoglobin (Hb), white blood cells (WBCs), platelets (PLTs), reticulocytes (Rets), lactate dehydrogenase (LDH), total bilirubin (TBIL), free Hb, haptoglobin and PNH clones, were analyzed. The clinical features of the patients are shown in Table 1.

**Table 1:** Clinical characteristics of all PNH patients

Characteristics	Sequencing group	Validation group
Total no. of patients	5	30
Gender M/F	3/2	19/11
Age Median (range)	38(16–62)	41(16–68)
Clinical classification n (%)		
Classical PNH	3(60.00)	22(73.33)
PNH-AA	2(40.00)	8(26.67)
History of thrombosis n (%)	0	11(36.67)
HGB (g/L)	76.60 ± 28.29	79.71 ± 23.97
Ret (%)	11.79 ± 7.44	8.285 ± 5.589
WBC (*109/L)	7.49 ± 3.53	5.47 ± 3.29
PLT (*109/L)	149.00 ± 159.20	116.00 ± 95.03
TBIL (umol/L)	24.48 ± 14.69	34.53 ± 18.59
LDH (U/L)	2236.00 ± 1498.0	1543.00 ± 1181.0
Granulocyte CD59^−^ (%)	96.41 ± 2.458	74.90 ± 24.39
Erythrocyte CD59^−^ (%)	74.12 ± 21.42	47.50 ± 31.77

Our study conformed to the “International ethical guidelines for biomedical research involving human subjects (2002),” which were developed by the Council for International Organizations of Medical Sciences (CIOMS) in collaboration with the World Health Organization (WHO), and was approved by the Ethical Committee of Tianjin Medical University.

### Cell sorting by flow cytometry

**Fig. 1:**
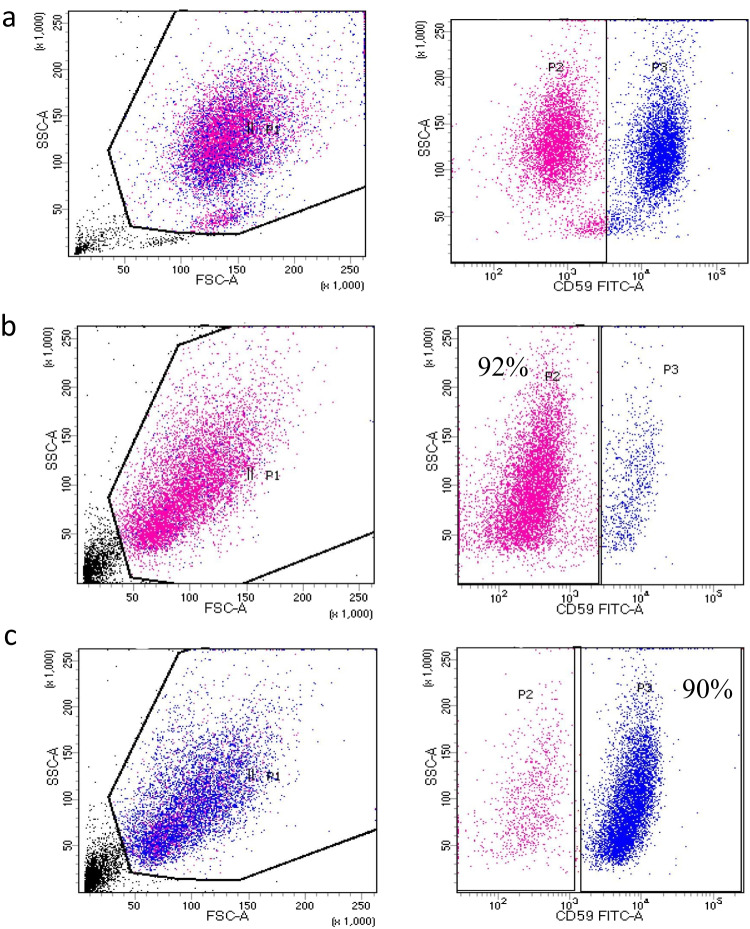
**a** The cells of PNH patients were sorted by flow cytometry to obtain CD59^−^ and CD59^+^ granulocytes and monocytes. **b** Purity of CD59^−^ granulocytes and monocytes after sorting. **c** Purity of the CD59^+^ granulocytes and monocytes after sorting. The sorting purity was greater than 90%

Ten milliliters of peripheral blood was collected from PNH patients into an ethylenediaminetetraacetic acid (EDTA) anticoagulant tube. First, the supernatants were discarded after centrifugation (3 g, 5 min), and the remaining blood was separated into five centrifuge tubes. After 15 min, the erythrocyte lysate was centrifuged again and washed with PBS. Anti-CD59 FITC (BD PharMingen, USA) was added and incubated for 20 min in a dark room. Furthermore, to obtain CD59^−^ and CD59^+^ granulocytes and monocytes, PNH clones were sorted by flow cytometry (BD FACSAria II, USA). The separation purity was greater than 90% (Fig. 1a, b, c).

### High-throughput sequencing

After obtaining CD59^−^ and CD59^+^ granulocytes and monocytes from PNH patients, total RNA was extracted. RNA-seq analysis was performed by Beijing Novogene Bioinformatics Technology Co., Ltd. (supplementary information 1).

### qRT‐PCR

Total RNA was extracted from CD59^−^ cells with the RNeasy kit (Takara Bio Inc., RR420A, Japan). cDNA was synthesized from 1 μg of total RNA using a reverse transcription kit (Takara, Japan) and purified with the QIAquick PCR Purification Kit (Qiagen). Quantitative PCR (qRT‒PCR) was performed in duplicate with a Quanti-Tect SYBR Green PCR Kit (Takara, Tli RNaseH Plus) on a Light Cycler 1.5 Real-time PCR machine (Roche, Indianapolis, IN, USA). Specific primers were designed to amplify cross-exon fragments of genes that included TAB2, TLR4, LYN, CFLAR, TNFAIP3, PTGS2, TRIM25, CXCL8, LINC01002 and FAM157C, and the primer sequences are detailed in Table 2. BIO-RAD CFX MANAGER software was used to analyze the melting curve and the amplification curve (quantitative curve), and the Ct values of each group were read. The relative quantitative multiplier of each group (relative fold, RF) was expressed as the 2 − ΔΔCt value and used for statistical analysis.

**Table 2:** Gene primer sequences

Gene	Forward	Reverse	Annealing temperature℃
LINC01002	TCCAGTCAGCCTCTACAGACCAAG	GATACGATGGAGCTGTGCCTGTG	57.0
FAM157C	AAGACGGAGCAGCACAGTCATTC	TGTTCGGACAGTTACACGCCATG	60.8
CFLAR	GCTGATGGCAGAGATTGGTGAGG	TCCAACTCAACCACAAGGTCCAAG	57.1
LYN	AGGCTCTACGCTGTGGTCACC	TTGCCACCTTCATCGCTCTTCAG	56.0
CXCL8	TCTCTTGGCAGCCTTCCTGA	TTTCTGTGTTGGCGCAGTGT	57.1
TNFAIP3	CTGCTGGCTGCCTGTCTCAAG	GTTCTGGAACCTGGACGCTGTG	56.5
PTGS2	TGGTCTGGTGCCTGGTCTGATG	CCTGCTTGTCTGGAACAACTGCTC	57.1
TLR4	GAGGCAGCTCTTGGTGGAAGTTG	CAAGCACACTGAGGACCGACAC	56.0
TRIM25	CTGGTGCGTGGAGTGGTTCAAC	TGTCGGCAACAGCGAAGAAGATG	56.5
TAB2	ACCTCCAGCACTTCCTCTTCAGTC	TGTTCATCTCCTGTGGCAGCATTC	56.0
GAPDH	CAGGAGGCATTGCTGATGAT	GAAGGCTGGGGCTCATTT	

### Cell line and cell culture

Cas9- and sgRNA-overexpressing lentiviruses were constructed to infect THP-1 cells, and GFP (Cas9 vector)- and mCherry (sgRNA vector)-positive cells were then screened by flow cytometry. The monoclonal cells were selected and amplified after identification, and finally, the PIGA gene-knockout (PIGA-KO) monoclonal cell line was obtained. PIGA-KO-THP-1 cells were cultured in RPMI-1640 + 10% fetal bovine serum (Gibco) + 1% double antibody. PIGA-KO cell clones were established based on mutations as assessed by PCR, gene sequencing and their phenotypes (loss of FLAER expression and CD59 positive expression). The cells were cultured in a constant temperature (37 °C) incubator in 5% CO_2_ and were passaged at a ratio of 1:3 every 48 h.

### Lentivirus transfection

A lentivirus vector expressing an shRNA targeting FAM157C was transfected into PIGA-Ko-THP-1 cells for 72 h. The cells were divided into three groups: the control group, the empty virus group, and the FAM157C knockdown group. A total of 5 × 10^4^ cells were seeded in 24-well plates, in a final volume of 500 µl, and incubated at 37 °C in 5% CO_2_ for 72 h. The transfection rate and knockdown rate were evaluated by flow cytometry and qRT‒PCR. These cells were used to obtain stable transfectants.

### CCK-8 assay

For the measurement of cell proliferation, we utilized the CCK-8 kit (Dojindo Laboratories) to perform CCK-8 assays. Cells were seeded in a 96-well plate and incubated in CCK-8 solution for 4 h at 37 °C in a 5% CO_2_ incubator. Then, the absorbance was measured at a wavelength of 450 nm.

### Cell apoptosis assay

Apoptosis was determined by the translocation of phosphatidylserine to the cell surface using an Annexin V-PE and 7-ADD apoptosis detection kit (BD PMG). PIGA-KO-THP-1 cells with stable knockdown of lncRNA FAM157C and the negative control cells were harvested and washed twice in cold PBS; then, the cells were resuspended in Annexin V-PE and 7-ADD and incubated for 30 min in the dark. Cell apoptosis was analyzed by using Cell Quest software on a flow cytometer (Beckman).

### Cell cycle analysis

The cells were first harvested after 72 h of transfection, and the cell suspension was then digested. Then, the cells were fixed with ethanol (70%) for 4 h at 4 °C, and the supernatants were discarded, followed by incubation with an RNA enzyme solution containing iodide (PI, BD PMG). After the cells were washed with PBS three times, the cell cycle was analyzed by using a flow cytometer (Beckman), and data analysis was conducted by Kaluza. The experiment was repeated three times.

### Statistical analysis

GraphPad Prism 5 statistical software was used for statistical analysis. The results are expressed as the mean ± standard deviation. The independent sample mean comparison was performed using the *t* test (for data with normal distribution) and nonparametric test (for data without normal distribution). Spearman’s correlation analysis was used to evaluate the association between qualitative variables. A value of *P* < 0.05 was considered statistically significant.

## Results

### Some abnormally expressed lncRNAs and mRNAs in PNH clones were identified by high-throughput sequencing

**Fig. 2:**
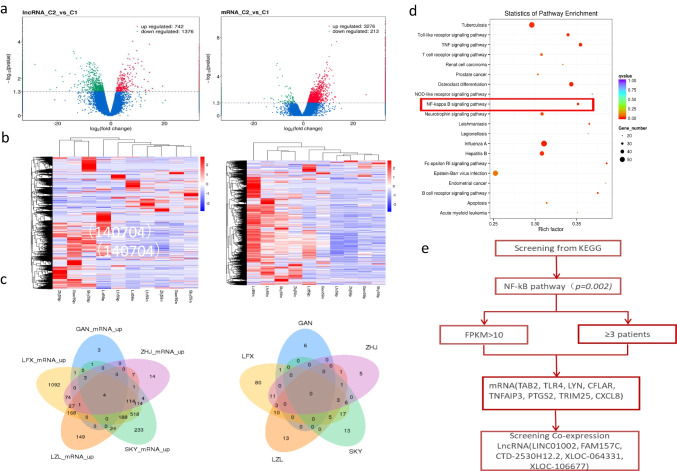
**a** Volcano map of differentially expressed lncRNAs and mRNAs. C1 represents CD59^+^ cells, and C2 represents CD59^−^ cells. **b** Heatmap of differentially expressed lncRNAs and mRNAs. **c** Venn diagrams of upregulated lncRNAs and mRNAs in 5 PNH patients. Four mRNAs were expressed in 5 patients, 118 mRNAs were expressed in 4 patients, 335 mRNAs were expressed in 3 patients, 3 lncRNAs were expressed in 4 patients, and 15 lncRNAs were expressed in 3 patients. **d** A scatter plot is a graphical representation of the KEGG enrichment analysis results. In this figure, KEGG enrichment was measured by rich factor, *q*-value and the number of genes enriched in this pathway. The greater the Rich factor is, the greater the degree of enrichment. The *q*-value is the *p* value after multiple hypothesis testing and correction. The value range of the *q* value is [0,1]. The closer the value is to zero, the more significant the enrichment. **e** Screening of mRNAs and lncRNAs related to the NF-κB pathway

To perform transcriptome profiling of CD59^−^ cells and CD59^+^ cells in PNH patients, high-throughput sequencing was performed in CD59^−^ and CD59^+^ granulocytes and monocytes from 5 PNH patients. Transcription analysis revealed 742 upregulated and 1376 downregulated lncRNAs and 3276 upregulated and 213 downregulated mRNAs (Fig. 2a, b).

The 173 upregulated mRNAs with fragments per kilobase of exon model per million mapped fragments (FPKM) > 5 in at least 3 patients were identified. Then, we identified 30 mRNAs related to proliferation, apoptosis and thrombosis (supplementary information 2). The 7 upregulated lncRNAs with FPKM > 5 in at least 3 patients were identified (supplementary information 2), including MALAT1, RP11-22N19.2, LUCAT1, FAM157C, CTD-2530H12.2, RP4-669L17.10 and RP13-20L14.10. The expression of the upregulated mRNAs and lncRNAs in 5 PNH patients is shown by Venn diagrams (Fig. 2c).

Meanwhile, in order to screen the lncRNAs related with proliferation, we also focused on the analysis of pathways which are associated with proliferation. According to KEGG pathway enrichment analysis, putative gene network interactions of the differentially expressed mRNAs were significantly enriched for the TNF signaling pathway, NF-κB signaling pathway, and Neurotrophin signaling pathway. We focused on the proliferation-related NF-kB pathway (Fig. 2d). The differentially expressed mRNAs in this pathway, with FPKM > 10 in at least 3 patients, were selected. Then the analysis of lncRNAs related to mRNAs by coexpression was performed to identify their upstream regulatory lncRNAs. Finally, a total of 8 mRNAs (TAB2, TLR4, LYN, CFLAR, TNFAIP3, PTGS2, TRIM25, and CXCL8) were identified, and upstream regulatory lncRNAs (LINC01002, FAM157C, CTD-2530H12.2, XLOC-064331, and XLOC-106677) were identified by interaction analysis (Fig. 2e). The 8 mRNAs and 5 lncRNAs were upregulated in CD59^−^ granulocytes and monocytes. Fortunately, we found the only same lncRNA, FAM157C, screened by two methods. Next, we tried to identify the expressions of these lncRNAs in more PNH patients by qRT-PCR.

### LncRNA FAM157C was verified to be upregulated by qRT‒PCR and its expression correlated with indicators of hemolysis

**Fig. 3:**
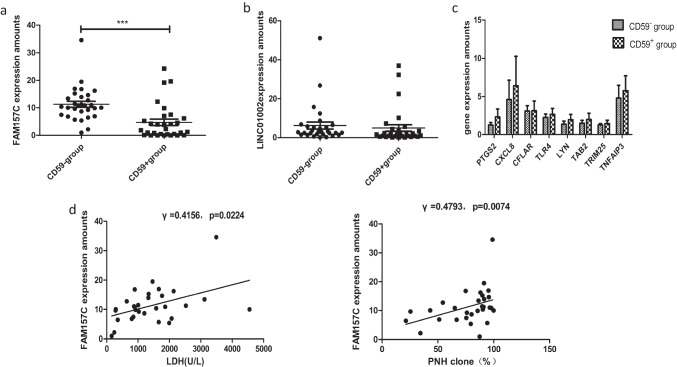
**a** The expression of FAM157C in 30 PNH patients; **b** the expression of LINC01002 in 30 PNH patients; **c.** the expression of mRNAs in 30 PNH patients; **d** analysis of the correlation between FAM157C expression and clinical data. The expression level of FAM157C was positively correlated with LDH levels and CD59^−^ granulated and monocyte cell ratios

The expression levels of some abnormally expressed lncRNAs and mRNAs in 30 PNH patients were measured by qRT‒PCR. As the qRT‒PCR results showed, lncRNA FAM157C (11.530 ± 6.628) expression in the PNH clone was consistently higher than that (5.482 ± 6.785, *p* = 0.0055) in CD59^+^ cells from 30 PNH patients (Fig. 3a). LncRNA LINC01002 expression was 6.118 ± 10.08 in the PNH clone, which was not significantly different from that in CD59^+^ cells (4.883 ± 9.208, *p* = 0.6374) (Fig. 3b). There were no significant differences in mRNA expression (Fig. 3c, Table 3).

**Table 3:** Comparison of mRNAs expression

mRNA	CD59^−^ granulocytes and monocytes	CD59^+^ granulocytes and monocytes	n	*P* value
LYN	1.373 ± 1.676	1.957 ± 2.956	30	0.4679
TLR4	2.256 ± 1.928	6.213 ± 15.84	30	0.3001
TAB2	1.496 ± 1.569	2.989 ± 5.373	30	0.2821
PTGS2	1.963 ± 2.491	2.336 ± 3.475	30	0.4462
CXCL8	4.606 ± 7.600	6.423 ± 11.55	30	0.2228
CFLAR	3.091 ± 2.126	3.128 ± 3.872	30	0.9662
TRIM25	1.253 ± 0.8713	2.846 ± 5.872	30	0.2677
TNFAIP3	3.426 ± 4.189	5.769 ± 8.517	30	0.1095

The correlation between the expression of lncRNA FAM157C and the clinical indexes (routine blood, reticulocyte, and hemolysis indicators) was analyzed. The higher expression of FAM157C was positively correlated with LDH levels and (*r* = 0.4156, *p* = 0.0224) CD59^−^ granulated and monocyte cell ratios (*r* = 0.4793, *p* = 0.0074) (Fig. 3d).

### Knockdown of lncRNA FAM157C inhibited the proliferation of PIGA-KO THP-1 cells

**Fig. 4:**
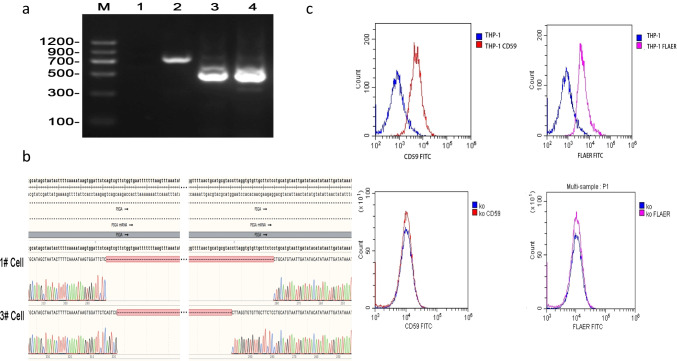
**a** PCR identification of PIGA-KO-THP-1 monoclonal cells. M: DL-1200, Lane-2: Negative Control, Lane-3: THP-1 WT Cell, Lane-4: #1 THP-1 KO cell, Lane-5: #3 THP-1 KO cell. **b** Gene sequencing results of PIGA-KO-THP-1 monoclonal cells. #1 and #3 cells: THP-1 KO cells. **c** Surface expression of GPI-APs on THP-1 cells and PIGA-KO-THP-1 cells. Experimental cells were stained with FLAER (purple lines) and anti-CD59 (red lines) antibodies. The negative staining controls (blue lines) for FLAER and anti-CD59 were buffer alone and isotype matched monoclonal antibody, respectively. The expression of GPI-APs by PIGA-KO cells was lost

**Fig. 5:**
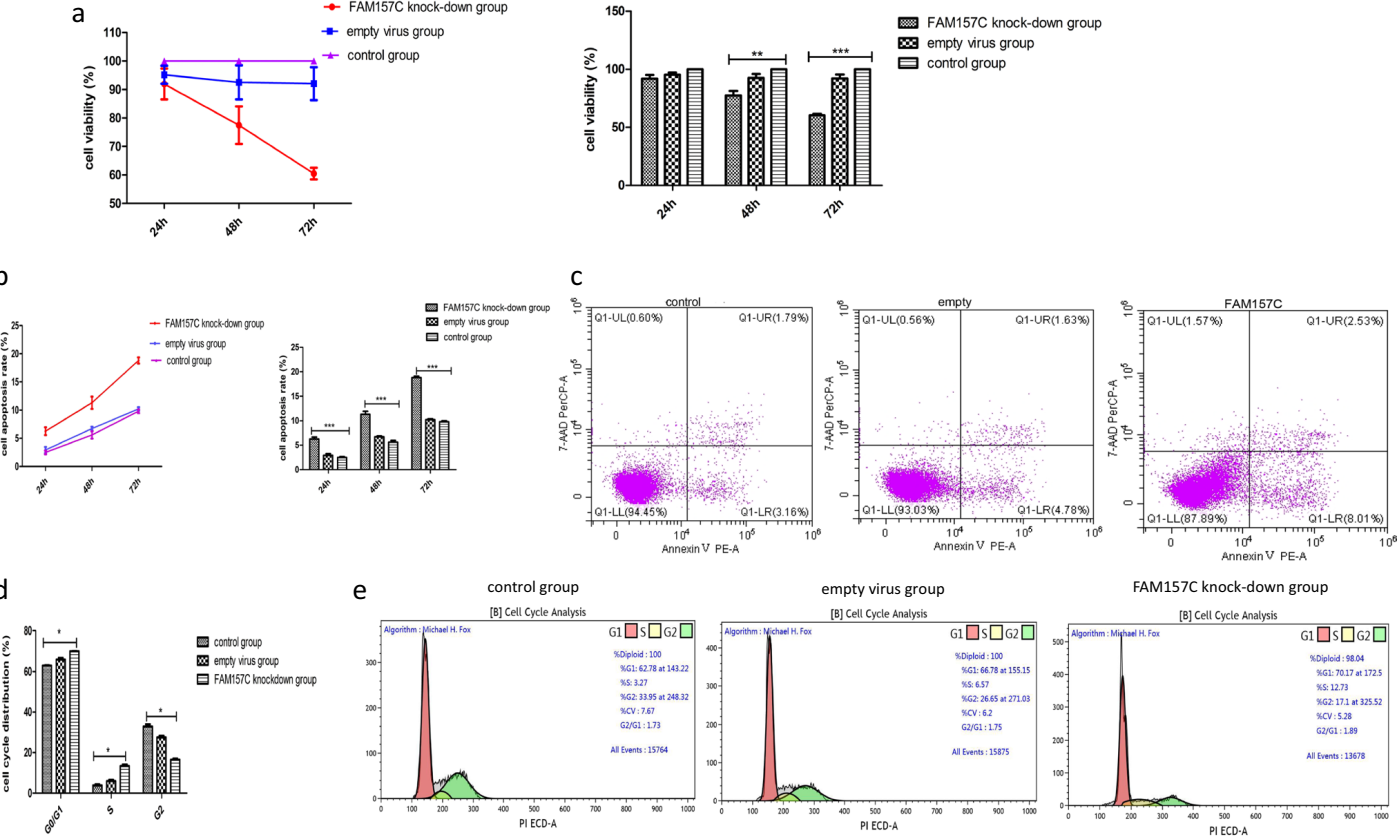
**a** Significantly decreased proliferation was detected after FAM157C knockdown compared with the control group and empty virus transfection group; **b** significantly increased cell apoptosis rate was observed after FAM157C knockdown compared with the control group and empty virus transfection group; **c** the cell apoptosis rate was examined by flow cytometry; **d** the cell cycle phase assay after transfection. The percentage of postreplicating cells in the G2 phase in the FAM157C knockdown group was significantly reduced, while the percentage of cells in the G0/G1 phase and S phase was significantly increased compared to the control group and empty virus transfection group. **e** The cell cycle progression was examined by flow cytometry

The verification results from PIGA-KO-THP-1 cells are shown in Fig. 4a, b, c. LncRNA FAM157C was knocked down by lentivirus; the transfection rate was 70%, and the knockdown rate was 90%. The cell viability (%) of the control group, empty virus group and lncRNA FAM157C knockdown group was 100 ± 0, 95.20 ± 3.178 and 91.93 ± 5.423, respectively, after 24 h of transfection. There was no significant difference between the three groups (*p* = 0.1807). At 48 h and 72 h, the cell viability of the three groups was 100 ± 0 vs. 93.75 ± 5.995 vs. 77.49 ± 6.597 and 100 ± 0 vs. 92.795 ± 5.802 vs. 60.47 ± 2.059, respectively. The viability of the FAM157C knockdown group was significantly lower than those of the control and empty virus groups (*p* = 0.0275, *p* = 0.0388, *p* = 0.0009, *p* = 0.0052) (Fig. 5a).

Meanwhile, the apoptosis rate increased after transfection with FAM157C-targeting shRNA. The apoptosis rate of the FAM157C knockdown group (6.256 ± 0.5453)% was significantly higher than that of the control group (2.483 ± 0.3083)% and empty virus group (2.926 ± 0.5517)% (*p* = 0.0138, 0.0066) at 24 h. There was no significant difference between the empty virus group and the control group (*p* = 0.0899). Similar results were found at 48 h and 72 h. The apoptosis rates of the three groups were 5.593 ± 0.6400%, 6.723 ± 0.3256%, and 11.30 ± 1.075% at 48 h (*p* = 0.0137, *p* = 0.0217) and 9.797 ± 0.3235%, 10.21 ± 0.3005%, and 18.81 ± 0.5363% at 72 h (*p* = 0.0012, *p* = 0.00087) (Fig. 5b, c).

We also observed cell cycle progression, and the results showed that the proportion of cells in the G0/G1 phase and S phase increased while the proportion of cells in the G2 phase decreased, indicating that the cells were arrested at the G0/G1 phase and S phase. The cells in the G0/G1 phase in the control group, empty virus group and FAM157C knockdown group accounted for 62.98 ± 1.513%, 65.95 ± 1.174% and 70.00 ± 0.2404%, those in the S phase accounted for 3.825 ± 0.7849%, 5.920 ± 0.9192% and 13.47 ± 1.039%, and those in the G2 phase accounted for 32.81 ± 1.612%, 27.47 ± 1.160% and 16.54 ± 0.7990% after transfection, and these results were significantly different (*p* = 0.0269, *p* = 0.0198, *p* = 0.0145). (Fig. 5d, e).

## Discussion

In recent years, studies have found that PIG-A mutations can also be observed in normal people, accounting for approximately 10%. However, no PNH clones or proliferation have been found, and there are no clinical symptoms of PNH in these individuals [14]. In Shin TH et al. 2019, data were collected from the PNH macaque model in which CRISPR/Cas9 technology was used to generate a model of hematologic disease based on the near phylogenetic/functional similarity between macaques and humans; the results showed that there was no intrinsic clonal amplification of PNH-HSPCs [15]. At present, studies have reported that immune-escape characteristics [16–18], anti-apoptotic properties [19, 20], and second gene mutations [21–23] may be involved in the amplification of PNH clones. T-lymphocyte attack GPI^+^ HSCs, and GPI^−^ HSCs are less vulnerable to attack. Some research has found that PNH clones are protected from NK/T effector cells due to the lack of GPI-anchored cytomegalovirus ul-16 binding protein (ULBPs) or CD1d restriction [24, 25]. CD109 has been reported to be a protein in GPI-APs, a TGF coreceptor, which plays a key role in inhibiting TGF-signal-mediated erythrocyte differentiation. The lack of CD109 expression may make PIGA-mutated HSPCs more sensitive to TGF-β, leading to easier differentiation of mutant erythroid progenitors into mature erythrocytes [26]. Studies have found that PNH clones have anti-apoptotic properties. In addition, Bcl-2, Bcl-XL, Bag-1, McL-1 and other anti-apoptotic genes were significantly increased in PNH patients and played an important role in the anti-apoptotic process [27]. The researchers also found that PIG-A mutations disrupt lipid raft formation of the cell membrane, which changes passivation to promote apoptosis signals or inhibit growth [28]. The theory of secondary genetic mutations was first reported in the 1970s [21]. Mutations in additional genes, such as HMGA2 [29, 30], WT1 [31], TET2 [32] and RBPJ [33], have been reported in PNH patients. Most patients with PNH can carry additional mutations, and these mutations are secondary insults. PIGA mutation is the initial mutation, the nature of PNH is a single gene disease, and its clinical manifestations are mainly determined by PIGA mutations rather than myeloid gene mutations [34]. However, various theories cannot explain all the pathological mechanisms, and PNH clones must be involved in other mechanisms to result in a proliferation advantage in patients.

Although lncRNAs do not encode proteins, they play an important role in cell proliferation and differentiation and are widely studied in oncologic diseases. The roles of lncRNA have been studied not only in solid tumors but also in nonsolid tumors and even autoimmune diseases. The lncRNAs LOC101928834, H19, WT1-AS, TCL6, LEF1-AS1, EPB41L4A-AS1, PVT1, GAS5 and ZFAS were found to be relevant to myelodysplastic syndrome (MDS) pathogenesis and outcome [35, 36]. Many lncRNAs, such as MALAT1, GAS5, DLEU2, and H19, have been reported to be involved in the diagnosis and progression of multiple myeloma (MM) [37, 38]. The lncRNAs HOTAIR, lincRNA‐p21, lncRNA H19 and MALAT1 play important roles in the clinical diagnosis and progression of rheumatoid arthritis (RA) [39]. The mechanisms of action of lncRNAs are complex. LncRNAs can interact with DNA, RNA, or proteins. LncRNAs are involved in various pathways, including the p53, NF-κB, PI3K/AKT, and Notch pathways.

The PNH clone has biological characteristics similar to those of tumor cells to gain a proliferative advantage. In our study, the results of high-throughput sequencing in PNH patients showed a large number of differentially expressed lncRNAs and mRNAs, many of which were involved in cell proliferation, thrombosis, etc. Such results provided substantial information, and lncRNAs may play important roles in PNH clone proliferation. With verification, we found that the level of lncRNA FAM157C in primary PNH cells was significantly increased and positively correlated with hemolysis indicators. Hochsmann B et al. [40] knocked out the PIGA and PIGT genes in THP-1 cell lines and further proved that PIGA-PNH and PIGT-PNH had different cloning mechanisms and clinical manifestations. Therefore, we studied the function of FAM157C in the PIGA-KO THP-1 cell line. After knockdown of the FAM157C gene, the proliferation of PIGA-KO cells decreased. The results of our experiment suggested that the FAM157C gene may promote PNH clone proliferation.

The length of the FAM157C gene sequence is 961 bp. It is expressed in the bone marrow, spleen and other organs, but its function has not been reported in the literature. We further tried to overexpress the FAM157C gene but failed several times because it was too long for virus transfection. The mechanism underlying the function of FAM157C remains to be further studied.

Early this century, the use of the anti-C5 antibody eculizumab has changed the management of PNH patients and may further improve their life. Noval complement inhibitors, like ravulizumab, a long-term C5 inhibitor, and pegcetacoplan, a C3 inhibitor, have also been now licensed for PNH. New drugs like factor D and factor B inhibitors are under study. For eculizumab and ravulizumab, there are still some limitations, such as extravascular hemolysis, the need for lifelong medication, and substantial costs [41]. Pegcetacoplan is capable of blocking both intravascular and extravascular hemolysis, only breakthrough hemolysis is a potential complication [42].

Current prevalent approaches to lncRNA targeting include small interfering RNAs (siRNAs), antisense oligonucleotides (ASOs), clustered regularly interspaced short palindromic repeats (CRISPR), oligonucleotide therapeutics, and so on [43]. Although most of the studies on lncRNA targeting are still in the preclinical stage, targeting lncRNAs is an emerging concept and strategy. Identifying the most potential lncRNAs is the first step and the most important process.

In conclusion, lncRNA FAM157C was proven to promote PNH clone proliferation, and this is the first study to explore the role of lncRNAs in PNH. Fully understanding the role of lncRNAs involved in the pathogenesis of PNH may provide more new and accurate strategies in treatment. However, more studies are needed to further investigate the function of FAM157C in the future.

## Supplementary Information

Below is the link to the electronic supplementary material.

Supplementary file1 (DOC 44 KB)

Supplementary file2 (DOC 64 KB)

## Data Availability

The data that support the findings of this study are available from the corresponding author upon reasonable request.
