# Supplementary material for: Long noncoding RNA FAM157C contributes to clonal proliferation in paroxysmal nocturnal hemoglobinuria
Source: Ann Hematol. 2023 Jan 6;102(2):299–309. doi: 10.1007/s00277-022-05055-8 (PMC9889514; doi:10.1007/s00277-022-05055-8)
Supplement: Supplementary file 1 — Supplementary file1 (DOC 44 KB) [file 277_2022_5055_MOESM1_ESM.doc]

**Methods**

**Sample collection and preparation**

**RNA isolation, library preparation and sequencing**

**◇ RNA isolation**

**◇ RNA quantification and qualification (Novogene Experimental Department)**

RNA degradation and contamination was monitored on 1% agarose gels.

RNA purity was checked using the NanoPhotometer® spectrophotometer (IMPLEN, CA, USA).

RNA concentration was measured using Qubit® RNA Assay Kit in Qubit® 2.0 Flurometer (Life Technologies, CA, USA).

RNA integrity was assessed using the RNA Nano 6000 Assay Kit of the Bioanalyzer 2100 system (Agilent Technologies, CA, USA).

- **Library preparation for lncRNA sequencing (Novogene Experimental Department)**

A total amount of 3 μg RNA per sample was used as input material for the RNA sample preparations. Firstly, ribosomal RNA was removed by Epicentre Ribo-zero™ rRNA Removal Kit (Epicentre, USA), and rRNA free residue was cleaned up by ethanol precipitation. Subsequently, sequencing libraries were generated using the rRNAdepleted RNA by NEBNext® Ultra™ Directional RNA Library Prep Kit for Illumina®(NEB, USA) following manufacturer’s recommendations. Briefly, fragmentation was carried out using divalent cations under elevated temperature in NEBNext First Strand Synthesis Reaction Buffer(5X). First strand cDNA was synthesized using random hexamer primer and M-MuLV Reverse Transcriptase(RNaseH-). Second strand cDNA synthesis was subsequently performed using DNA Polymerase I and RNase H. In the reaction buffer, dNTPs with dTTP were replaced by dUTP. Remaining overhangs were converted into blunt ends via exonuclease/polymerase activities. After adenylation of 3’ ends of DNA fragments, NEBNext Adaptor with hairpin loop structure were ligated to prepare for hybridization. In order to select cDNA fragments of preferentially 150~200 bp in length, the library fragments were purified with AMPure XP system (Beckman Coulter, Beverly, USA). Then 3 μl USER Enzyme (NEB, USA) was used with size-selected, adaptor-ligated cDNA at 37° C for 15 min followed by 5 min at 95°C before PCR. Then PCR was performed with Phusion High-Fidelity DNA polymerase, Universal PCR primers and Index (X) Primer. At last, products were purified (AMPure XP system) and library quality was assessed on the Agilent Bioanalyzer 2100 system.

- **Clustering and sequencing (Novogene Experimental Department)**

The clustering of the index-coded samples was performed on a cBot Cluster Generation System using TruSeq PE Cluster Kit v3-cBot-HS (Illumia) according to the manufacturer’s instructions. After cluster generation, the libraries were sequenced on an Illumina Hiseq 4000 platform and 150 bp paired-end reads were generated. **Data analysis ( Novogene Gene Regulation Department )**

**◇ Quality control**

Raw data(raw reads) of fastq format were firstly processed through in-house perl scripts. In this step, clean data(clean reads) were obtained by removing reads containing adapter, reads on containing ploy-N and low quality reads from raw data. At the same time, Q20, Q30 and GC content of the clean data were calculated. All the down stream analyses were based on the clean data with high quality.

- **Mapping to the reference genome**

Reference genome and gene model annotation files were downloaded from genome website directly. Index of the reference genome was built using bowtie2 v2.2.8 and paired-end clean reads were aligned to the reference genome using HISAT2v2.0.4. HISAT2 was run with ‘--rna-strandness RF’, other parameters were set as default.

- **Transcriptome assembly**

The mapped reads of each sample were assembled by StringTie (v1.3.1) in a reference-based approach. StringTie uses a novel network flow algorithm as well as an optional de novo assembly step to assemble and quantitate fulllength transcripts representing multiple splice variants for each gene locus.

- **Coding potential analysis**

**CNCI**

CNCI (Coding-Non-Coding-Index) (v2) profiles adjoining nucleotide triplets to effectively distinguish protein-coding and non-coding sequences independent of known annotations. We use CNCI with default parameters.

**CPC**

CPC (Coding Potential Calculator) (0.9-r2) mainly through assess the extent and quality of the ORF in a transcript and search the sequences with known protein sequence database to clarify the coding and non-coding transcripts. We used the NCBI eukaryotes' protein database and set the e-value ‘1e-10’ in our analysis.

**Pfam-sca**

We translated each transcript in all three possible frames and used Pfam Scan (v1.3) to identify occurrence of any of the known protein family domains documented in the Pfam database (release 27; used both Pfam A and Pfam B). Any transcript with a Pfam hit would be excluded in following steps. Pfam searches use default parameters of -E 0.001 --domE 0.001.

**phyloCSF**

PhyloCSF (phylogenetic codon substitution frequency) (v20121028) examines evolutionary signatures characteristic to alignments of conserved coding regions, such as the high frequencies of synonymous codon substitutions and conservative amino acid substitutions, and the low frequencies of other missense and non-sense substitutions to distinguish protein-coding and non-coding transcripts. We build multi-species genome sequence alignments and run phyloCSF with default parameters. Transcripts predicted with coding potential by either/all of the four tools above were filtered out, and those without coding potential were our candidate set of LncRNAs.

- **Conservative analysis**

Phast (v1.3) is a software package contains much of statistical programs, most used in phylogenetic analysis, and phastCons is a conservation scoring and identificating program of conserved elements. We used phyloFit to compute phylogenetic models for conserved and non-conserved regions among species and then gave the model and HMM transition parameters to phyloP to compute a set of conservation scores of LncRNA and coding genes.

- **Target gene prediction**

**Cis role of target gene prediction**

Cis role is LncRNA acting on neighboring target genes. We searched coding genes 10k/100k upstream and downstream of LncRNA and then analyzed their function next.

**Trans role of target gene prediction**

Trans role is LncRNA to identify each other by the expression level. While there were no more than 25 samples, we calculated the expressed correlation between LncRNAs and coding genes with custom scripts; otherwise, we clustered the genes from different samples with WGCNA to search common expression modules and then analyzed their function through functional enrichment analysis.

- **Quantification of gene expression level**

Cuffdiff (v2.1.1) was used to calculate FPKMs of both lncRNAs and coding genes in each sample. Gene FPKMs were computed by summing the FPKMs of transcripts in each gene group. FPKM means fragments per kilo-base of exon per million fragments mapped, calculated based on the length of the fragments and reads count mapped to this fragment.

- **Differential expression analysis**

The Ballgown suite includes functions for interactive exploration of the transcriptome assembly, visualization of transcript structures and feature-specific abundances for each locus, and post-hoc annotation of assembled features to annotated features. Transcripts with an P-adjust <0.05 were assigned as differentially expressed.

Cuffdiff provides statistical routines for determining differential expression in digital transcript or gene expression data using a model based on the negative binomial distribution. Transcripts with an P-adjust <0.05 were assigned as differentially expressed.

- **GO and KEGG enrichment analysis**

Gene Ontology (GO) enrichment analysis of differentially expressed genes or LncRNA target genes were implemented by the GOseq R package, in which gene length bias was corrected. GO terms with corrected Pvalue less than 0.05 were considered significantly enriched by differential expressed genes. KEGG is a database resource for understanding high-level functions and utilities of the biological system, such as the cell, the organism and the ecosystem, from molecular-level information, especially large-scale molecular datasets generated by genome sequencing and other high-throughput experimental technologies (http://www.genome.jp/kegg/). We used KOBAS software to test the statistical enrichment of differential expression genes or LncRNA target genes in KEGG pathways.

- **PPI analysis**

PPI analysis of differentially expressed genes was based on the STRING database, which known and predicted Protein-Protein Interactions. For the species existing in the database, we construct the networks by extract the target gene list from the database; Otherwise, Blastx (v2.2.28) was used to align the target gene sequences to the selected reference protein sequences, and then the networks was built according to the known interaction of selected reference species.

- **Alternative splicing analysis**

Alternative splicing events were classified to 12 basic types by the software Asprofile v1.0. The number of AS events in each sample was estimated, separately.

- **SNP analysis**

Picard-tools v1.96 and samtools v0.1.18 were used to sort, mark duplicated reads and reorder the bam alignment results of each sample. GATK2 software was used to perform SNP calling.
