# Supplementary material for: Long noncoding RNA FAM157C contributes to clonal proliferation in paroxysmal nocturnal hemoglobinuria
Source: Ann Hematol. 2023 Jan 6;102(2):299–309. doi: 10.1007/s00277-022-05055-8 (PMC9889514; doi:10.1007/s00277-022-05055-8)
Supplement: Supplementary file 2 — Supplementary file2 (DOC 64 KB) [file 277_2022_5055_MOESM2_ESM.doc]

supplementary material 2: List of LncRNAs and mRNAs related to proliferation, apoptosis and thrombosis

| Gene ID | LncRNAs | | Description | C2_FPKM | C1_FPKM | log2(foldchange) | p value |
| --- | --- | --- | --- | --- | --- | --- | --- |
| ENSG00000251562.7 | MALAT1 | | metastasis associated lung adenocarcinoma transcript 1 | 175.9457856 | 34.919761 | 2.333015368 | 0.017900711 |
| ENSG00000273320.1 | RP11-22N19.2 | |  | 60.0194632 | 15.9855486 | 1.90866216 | 0.043508919 |
| ENSG00000248323.5 | LUCAT1 | | lung cancer associated transcript 1 | 21.0571056 | 4.5544432 | 2.20896055 | 0.028837389 |
| ENSG00000260528.3 | FAM157C | | family with sequence similarity 157 member C | 12.0434488 | 1.9197286 | 2.649274315 | 0.010310792 |
| ENSG00000254826.1 | CTD-2530H12.2 | |  | 9.3377056 | 1.7240268 | 2.437285903 | 0.034419508 |
| ENSG00000237094.11 | RP4-669L17.10 | |  | 5.7889236 | 0.772379 | 2.905914272 | 0.034878569 |
| ENSG00000279744.1 | RP13-20L14.10 | |  | 5.347006 | 0.3594232 | 3.894975856 | 0.004307905 |
|  | mRNAs | |  |  |  |  |  |
| ENSG00000180871 | CXCR2 | C-X-C motif chemokine receptor 2 | | 154.6407214 | 9.5702072 | 4.014226302 | 0.002429133 |
| ENSG00000077238 | IL4R | interleukin 4 receptor | | 17.53990460 | 3.54214620 | 2.30794524 | 0.04991873 |
| ENSG00000163739 | CXCL1 | C-X-C motif chemokine ligand 1 | | 15.76438500 | 3.02190020 | 2.38314097 | 0.01623132 |
| ENSG00000081237 | PTPRC | protein tyrosine phosphatase receptor type C | | 26.27931140 | 1.71169820 | 3.94042721 | 0.01096590 |
| ENSG00000173334 | TRIB1 | tribbles pseudokinase 1 | | 26.17910740 | 6.20820500 | 2.07616781 | 0.02434938 |
| ENSG00000169429 | CXCL8 | C-X-C motif chemokine ligand 8 | | 162.3738572 | 29.0056696 | 2.484912542 | 0.014424356 |
| ENSG00000135821 | GLUL | glutamate-ammonia ligase | | 95.20167980 | 11.93431560 | 2.99587120 | 0.03607390 |
| ENSG00000143226 | FCGR2A | Fc fragment of IgG receptor IIa | | 91.04397660 | 20.92131000 | 2.12159038 | 0.03303273 |
| ENSG00000125347 | IRF1 | interferon regulatory factor 1 | | 53.43530560 | 13.54967220 | 1.97953532 | 0.04484982 |
| ENSG00000115956 | PLEK | pleckstrin | | 33.93847540 | 7.75113200 | 2.13044283 | 0.02434756 |
| ENSG00000197081 | IGF2R | insulin like growth factor 2 receptor | | 23.70499740 | 5.31326740 | 2.15752001 | 0.03895071 |
| ENSG00000254087 | LYN | LYN proto-oncogene, Src family tyrosine kinase | | 20.79055920 | 2.32632460 | 3.15980424 | 0.00479006 |
| ENSG00000073756 | PTGS2 | prostaglandin-endoperoxide synthase 2 | | 18.49289840 | 3.12707600 | 2.56408517 | 0.00922148 |
| ENSG00000115590 | IL1R2 | interleukin 1 receptor type 2 | | 18.4240832 | 2.3503686 | 2.970633899 | 0.031113565 |
| ENSG00000197249 | SERPINA1 | serpin family A member 1 | | 28.8555134 | 6.3702154 | 2.179432939 | 0.02643933 |
| ENSG00000143384 | MCL1 | MCL1 apoptosis regulator, BCL2 family member | | 88.9015594 | 24.9678794 | 1.832135431 | 0.04704622 |
| ENSG00000123146 | ADGRE5 | adhesion G protein-coupled receptor E5 | | 48.9647406 | 9.3472116 | 2.389135283 | 0.028306868 |
| ENSG00000105835 | NAMPT | nicotinamide phosphoribosyltransferase | | 105.6284652 | 18.9038578 | 2.482246083 | 0.018151305 |
| ENSG00000163563 | MNDA | myeloid cell nuclear differentiation antigen | | 84.9128556 | 21.5566622 | 1.97784918 | 0.034582539 |
| ENSG00000103569 | AQP9 | aquaporin 9 | | 52.480405 | 10.01537 | 2.389563134 | 0.01873271 |
| ENSG00000137642 | SORL1 | sortilin related receptor 1 | | 45.0503394 | 9.0247356 | 2.319581404 | 0.024814519 |
| ENSG00000140575 | IQGAP1 | IQ motif containing GTPase activating protein 1 | | 34.1990464 | 7.8016304 | 2.13210854 | 0.033973567 |
| ENSG00000059804 | SLC2A3 | solute carrier family 2 member 3 | | 33.5308764 | 5.7538882 | 2.542881097 | 0.010642691 |
| ENSG00000115271 | GCA | grancalcin | | 31.993272 | 8.3017204 | 1.946286298 | 0.045215785 |
| ENSG00000100985 | MMP9 | matrix metallopeptidase 9 | | 29.293954 | 7.0366018 | 2.057652158 | 0.040533974 |
| ENSG00000105339 | DENND3 | DENN domain containing 3 | | 11.1790736 | 1.9070434 | 2.551391057 | 0.038989581 |
| ENSG00000188895 | MSL1 | MSL complex subunit 1 | | 11.389758 | 1.8326172 | 2.635759724 | 0.012418436 |
| ENSG00000189067 | LITAF | lipopolysaccharide induced TNF factor | | 11.1886882 | 1.6369952 | 2.772918903 | 0.012227707 |
| ENSG00000173559 | NABP1 | nucleic acid binding protein 1 | | 11.7908672 | 0.9997736 | 3.559924588 | 0.001661459 |
| ENSG00000132334 | PTPRE | protein tyrosine phosphatase receptor type E | | 12.7377982 | 2.1103496 | 2.593562001 | 0.03699557 |
